# Hallucinations during bipolar depressive episode treated with bupropion after alcohol withdrawal surgery: a case report

**DOI:** 10.3389/fpsyt.2026.1750892

**Published:** 2026-02-25

**Authors:** Xiangqi Kong, Lingzi Cao, Xiaoyuan Han, Ping Dong, Xu Chen

**Affiliations:** 1 Department of Psychiatry, School of Mental Health, Jining Medical College, Jining, Shandong, China; 2 Department of Psychosis, Xingtai People’s Hospital, Xingtai, Hebei, China; 3 Department of Psychology, Chengde Medical University, Chengde, Hebei, China; 4 School of Medical Technology, Xinxiang Medical University, Xinxiang, Henan, China; 5 Shandong Mental Health Center, Shandong University, Jinan, Shandong, China

**Keywords:** alcohol use disorder, alcohol withdrawal surgery, amygdala, bipolar II disorder, bupropion

## Abstract

Alcohol Use Disorder (AUD) is a prevalent condition often co-occurring with affective disorders. Bupropion, a medication used to treat affective disorders, acts by inhibiting dopamine (DA) and norepinephrine (NE) reuptake, potentially helping to alleviate emotional dysregulation. However, the risk of delirium in AUD patients at therapeutic doses is poorly understood, despite reports linking bupropion overdose to delirium. We report here a rare case of a 49-year-old Chinese male with AUD who developed mood disturbances and relapsed following alcohol detoxification surgery. He was diagnosed with 1. Bipolar II Disorder, Current Depressive Episode, Severe Without Psychotic Features; 2. Harmful Pattern of Alcohol Use. During inpatient treatment, the patient received a combination of medications, including bupropion. Delirium and behavioral disturbances appeared after bupropion initiation but resolved upon its discontinuation. These symptoms recurred when bupropion was reintroduced and resolved again after stopping the medication. After discharge, no further bupropion was used, and the patient remained symptom-free at a one-month follow-up. This case suggests that bupropion may trigger delirium in AUD patients with a history of alcohol withdrawal surgery, underscoring the importance of careful medication selection and personalized treatment in complex cases.

## 1 Introduction

AUD is a substance use disorder characterized by compulsive alcohol-seeking behavior, loss of control over intake, and negative emotional and physical withdrawal symptoms (1). Chronic excessive alcohol consumption can lead to serious health complications, including gastric ulcers, brain damage, alcoholic liver disease, and an increased risk of cancer (2–5). Bilateral stereotactic deep brain nucleus lesioning surgery has been validated as an effective treatment for AUD (6–9). AUD is frequently comorbid with other psychiatric disorders, such as bipolar disorder, schizophrenia, personality disorders, and other mental health conditions (10, 11).

In managing a depressive episode in a bipolar disorder patient, careful drug selection is critical to avoid worsening depressive symptoms. Bupropion is commonly used due to its favorable efficacy profile (12), as it inhibits DA and NE reuptake within the brain’s reward system (13). However, immediate-release (IR) bupropion, when taken in excessive doses, has been associated with adverse effects, including dizziness, tremors, nausea and/or vomiting, drowsiness, tachycardia, and hallucinations (14). In addition, there are previous case reports of acute psychosis following extended-release bupropion (ER) overdose (15). Bupropion-induced psychiatric reactions may be due to disturbances in the dopaminergic system (16), particularly in patients with a history of psychiatric symptoms, substance misuse, or drug interactions (17–21).

Delirium is defined as an acute or fluctuating change in mental status (22). Studies indicate that metabolic imbalances, polypharmacy, alcohol or substance use or dependence, and environmental stressors (e.g., isolation or restraint) collectively increase the risk of delirium (23).

Although it is unclear whether bupropion increases the risk of delirium in patients during treatment of depressive episodes in bipolar disorder who are treated after alcohol withdrawal surgery, we present the case of a 49-year-old Chinese male patient with bipolar disorder. This patient experienced frequent mood swings after undergoing alcohol detoxification surgery and relapsing multiple times. Following hospital admission due to depressive symptoms, he was diagnosed with 1. Bipolar II Disorder, Current Depressive Episode, Severe Without Psychotic Features; 2. Harmful Pattern of Alcohol Use. Bupropion was initiated for the management of his depressive episode. He subsequently developed delirium, including visual hallucinations, which subsided one day after discontinuation bupropion. Visual hallucinations re-emerged upon bupropion reintroduction. This case highlights a potential risk of delirium associated with bupropion treatment in AUD patients with a history of detoxification surgery.

## 2 Case presentation

The patient, a 49-year-old married male, began occasional alcohol use in 1987, which progressed to uncontrolled daily consumption by 2008, amounting to approximately 12 bottles of beer per day. In 2010, his alcohol intake increased to around 500 ml of high-proof spirits daily. He exhibited compulsive drinking behaviors and experienced typical alcohol withdrawal symptoms, including sweating, hand tremors, and anxiety—that were alleviated by resuming alcohol consumption. In 2016, he developed persistent depressive symptoms, including low mood and anhedonia. In July 2021, the patient was diagnosed with Alcohol-induced depressive disorder according to ICD-11 and underwent bilateral amygdala destruction surgery. Post-surgery, his mood improved, and he achieved abstinence from alcohol. However, in October 2021, he was admitted to the hospital for the first time due to depressed mood and behavioral disturbances. He was diagnosed with 1. Bipolar II Disorder, Current Depressive Episode, Severe Without Psychotic Features; 2. Harmful Pattern of Alcohol Use. During hospitalization, his Michigan Alcohol Screening Test (MAST) score was 15, indicating significant alcohol-related issues, and his Self-Rating Depression Scale (SDS) score was 60, suggesting moderate depression. Following treatment, his symptoms improved, and he was discharged. In December 2021, the patient resumed alcohol consumption, drinking 1–2 beers or 150 ml of spirits weekly. By April 2022, he exhibited symptoms of mood instability, irritability, pressured speech, and excessive spending. He was diagnosed with 1. Bipolar I Disorder, Current Episode Manic, Severe, Without Psychotic Features; 2. Harmful Pattern of Alcohol Use, and was hospitalized again. After discharge, the patient adhered to medication and maintained abstinence. However, by April 2023, he once again presented with depressive symptoms, including anhedonia, diminished speech, reduced activity, and a loss of interest, leading to further hospitalization. Notably, he had no history of hallucinations or delusions.

### 2.1 History of past illness

In 2020, he underwent common bile duct surgery for gallstones. In 2021, he received Bilateral Stereotactic Deep Brain Nucleus Lesioning Surgery for the treatment of AUD. The patient was hospitalized three times (in 2021, 2022, and 2023) for mood disorders and was stabilized on each occasion. The patient denies a history of infectious diseases (e.g., hepatitis, tuberculosis, malaria), chronic conditions (e.g., hypertension, heart disease), or significant neurological insults such as poisoning, high fever, coma, seizures, or traumatic brain injury.

### 2.2 Family history

Psychiatric abnormalities were reported in his maternal grandfather, paternal aunt, maternal aunt, and maternal cousin. Both his paternal aunt and maternal grandfather died by suicide.

### 2.3 Physical and laboratory examinations

Vital signs, laboratory tests, and neuroimaging were systematically assessed around symptom onset and post-symptom periods. Key abnormalities included neutropenia, elevated lymphocyte percentage, increased mean platelet volume, elevated total bile acids, and elevated homocysteine. Electrolytes, liver and renal function, glycated hemoglobin, and thyroid function remained within normal limits. Neuroimaging (brain MRI including T1WI, T2WI, T2-FLAIR, and DWI) showed only postoperative changes, with no new structural abnormalities. Detailed results at each time point are summarized in **Table 1**.

**Table 1:** Clinical and laboratory evaluation at admission and around delirium onset.

Time point	Assessment/test	Key findings	Reference range
Symptom onset	Vital signs	Within normal limits	—
Symptom onset	Complete blood count (CBC)	WBC 10.57 ×10^9^/L	WBC 4–10 ×10^9^/L
		RBC 3.95 ×10¹²/L,	RBC 3.5–5 ×10¹²/L
		HGB 129 g/L	HGB 120–160 g/L
		PLT 94 ×10^9^/L	PLT 100–300 ×10^9^/L
Symptom onset	Serum electrolytes	Na 143 mmol/L	Na 135–145 mmol/L
		K 4.03 mmol/L	K 3.5–5 mmol/L
		Cl 108 mmol/L	Cl 95–109 mmol/L
		Ca 2.22 mmol/L	Ca 2.2–2.8 mmol/L
		Mg 0.85 mmol/L	Mg 0.7–1.1 mmol/L
		P 0.79 mmol/L	P 0.96–2.1 mmol/L
Symptom onset	Neuroimaging (Brain MRI: T1WI, T2WI, T2-FLAIR, and DWI)	MRI showed postoperative changes consistent with prior stereotactic lesioning, with multiple symmetric lesions involving the bilateral basal ganglia, amygdala, and periventricular regions adjacent to the anterior horns of the lateral ventricles. No ventricular enlargement or midline shift was observed.	—
Post-symptom	CBC	HGB 119 g/L	HGB 120–160 g/L
		HCT 33.9%	HCT 37–52%
		MCH 32.7 pg	MCH 26–31 pg
		PLT 86 ×10^9^/L	PLT 100–300 ×10^9^/L
		NEUT% 76.8%	NEUT% 50–70%
		LYM% 11.9%	LYM% 20–40%
		MONO% 10.5%	MONO% 3–10%
		CRP 17.46 mg/L	CRP 0–10 mg/L

At symptom onset, vital signs were normal, with mild hematologic and electrolyte abnormalities and no acute neuroimaging findings. On the third day after symptom onset (post-symptoms), thrombocytopenia and inflammatory changes persisted without severe metabolic or organ dysfunction.

### 2.4 Further diagnostic workup

The patient recently underwent a comprehensive psychiatric assessment. Assessment tools included the Chinese version of the 24-item Hamilton Depression Rating Scale (HAMD), on which the patient scored 35, indicating severe depressive symptoms. The Hamilton Anxiety Rating Scale (HAMA) score was 6, suggesting an absence of anxiety symptoms. The Positive and Negative Syndrome Scale (PANSS) score was 30, reflecting relatively mild psychopathological symptoms.

The patient also completed self-assessment tools: the Beck Depression Inventory (BDI-20) score was 22, indicating moderate depressive symptoms; the Zung Self-Rating Anxiety Scale (SAS) score was 36, showing no anxiety symptoms. Additionally, the Symptom Checklist-90 (SCL-90) yielded a total score of 171, indicating mild psychological distress.

### 2.5 Final diagnosis

Based on a comprehensive analysis of the patient’s mood fluctuations, episode patterns, and functional status, a differential diagnosis of schizophrenia is warranted. Schizophrenia is characterized by primary thought disorders and affective blunting, where mood disturbances are typically secondary. It often presents with pronounced disorganization of mental functions, either in an episodic or progressive course, with residual psychotic features persisting during remission.

In contrast, this patient’s depressive mood appears as a primary symptom, with relatively normal functioning during inter-episode periods, and lacks prominent and persistent psychotic features such as hallucinations or delusions. Additionally, the patient has a long-term or frequent history of alcohol use, with significant impairments in physical, psychological, and social functioning following consumption.

Considering the patient’s history of alcohol use and mood symptoms, and following the criteria outlined in the International Classification of Diseases, 11th Revision (ICD-11) (24), the patient was diagnosed with: 1. Bipolar II Disorder, Current Depressive Episode, Severe Without Psychotic Features; 2. Harmful Pattern of Alcohol Use.

### 2.6 Treatment

On the first day of admission, based on the patient’s clinical presentation and diagnostic findings, the following pharmacological treatment plan was established: sodium valproate (0.4 g, three times daily), paliperidone extended-release (3 mg, once daily), escitalopram (5 mg, twice daily), and quetiapine (25 mg, once daily at bedtime for clarity). On the second day, bupropion (75 mg, twice daily) was added to further improve mood, and escitalopram was discontinued. By the eighth day, paliperidone was stopped, and olanzapine (2.5 mg, once daily at bedtime for clarity) was introduced. On the tenth day, the dose of bupropion was increased to 150 mg twice daily. Subsequently, on the eleventh day, olanzapine was increased to 5 mg once daily at bedtime for clarity.

However, on the seventeenth day, the patient exhibited unusual behaviors, such as pulling at doors, pacing in the hall, and reporting visual hallucinations, such as seeing bugs on the floor and cockroach eggs on a glass. The psychiatric evaluation revealed a delayed response in social interactions, intact person orientation but impaired time and place orientation, disorganized associative thinking, repetitive finger movements, pathological volition, and a lack of self-awareness, with hallucinations persisting through the night. The patient’s acute symptoms—including impaired attention, altered level of consciousness, disorientation to time and place, and intra-day symptom fluctuation—were consistent with a diagnosis of Delirium according to ICD-11 criteria, with a clear temporal association with recent drug exposure. Given the potential link between treatment adjustments and delirium onset, olanzapine and bupropion were promptly discontinued. The patient received intravenous citicoline sodium (0.50 g in 250 ml of 0.9% sodium chloride solution) as part of the standard clinical management. Laboratory analyses, including blood counts and biochemical levels, revealed no significant electrolyte imbalances. By the eighteenth day, the hallucinations had resolved.

On the twentieth day, escitalopram was reintroduced at 5 mg once daily. To further investigate the cause of delirium, bupropion was re-administered (150 mg, twice daily) on the twenty-fifth day under the supervision of a physician, with informed consent from the patient and family. During the supervised bupropion re-challenge, the patient was closely monitored in an inpatient setting with regular assessments of mental status and vital signs. Predefined stopping criteria were applied, and bupropion was discontinued immediately upon recurrence of acute neuropsychiatric symptoms, including confusion, impaired attention, and behavioral disorganization, to ensure patient safety. Unfortunately, visual hallucinations re-emerged on the twenty-sixth day. Discontinuation of bupropion on the twenty-seventh day led to the cessation of these hallucinations.

Following 30 days of treatment, the patient’s scores on the HAMD and HAMA had dropped to 7 and 2, respectively, indicating resolution of depressive symptoms, with no recurrence of visual hallucinations. At discharge, the patient reported a significant improvement in depressive symptoms. The patient’s score of 5 on the Chinese version of the Naranjo Adverse Drug Reaction Probability Scale (25) suggested a probable adverse reaction to bupropion, supporting the decision to discontinue it.

### 2.7 Outcome and follow-up

At the one-month follow-up, the patient demonstrated strong adherence to the medication regimen and maintained regular, prescribed use of medications outside the hospital. The patient reported good tolerance to the prescribed drugs, stable physical and mental health, and no recurrence of delirium or adverse drug reactions. The patient’s score on the HAMD had further decreased to 6, and the HAMA score to 4, indicating improvements in both depressive and anxiety symptoms. The patient resumed normal daily activities and work and actively engaged in preferred hobbies, reflecting an enhanced quality of life. The timeline of treatment and adverse reaction is illustrated in **Table 2**.

**Table 2:** Detailed medication administration during hospitalization.

Start date	Medication	Dose	Route	Frequency	Stop date
2023/06/06	Valproate	0.4 g	Oral	Three times daily	2023/07/02
2023/06/06	Paliperidone ER	3 mg	Oral	Once daily	2023/06/13
2023/06/06	Buspirone	5 mg	Oral	Three times daily	2023/06/13
2023/06/06	Escitalopram	5 mg	Oral	Twice daily	2023/06/25
2023/06/06	Quetiapine	25 mg	Oral	Once daily at bedtime	2023/06/08
2023/06/07	Bupropion	75 mg	Oral	Twice daily	2023/06/15
2023/06/08	Quetiapine	100 mg	Oral	Once daily at bedtime	2023/06/12
2023/06/12	Quetiapine	0.2 g	Oral	Once daily at bedtime	2023/06/19
2023/06/13	Buspirone	10 mg	Oral	Three times daily	2023/06/21
2023/06/13	Olanzapine	2.5 mg	Oral	Once daily at bedtime	2023/06/16
2023/06/15	Quetiapine	0.1 g	Oral	Once daily	2023/06/19
2023/06/15	Bupropion ER	0.15 g	Oral	Twice daily	2023/06/22
2023/06/16	Olanzapine	5 mg	Oral	Once daily at bedtime	2023/06/22
2023/06/19	Quetiapine	0.2 g	Oral	Twice daily	2023/06/21
2023/06/22	Lorazepam	1 mg	Oral	Once daily at bedtime	2023/06/30
2023/06/23	Citicoline sodium	0.5 g	Intravenous	Once daily	2023/07/02
2023/06/23	0.9% Sodium Chloride	250 ml	Intravenous	Once daily	2023/07/02
2023/06/25	Escitalopram	5 mg	Oral	Once daily	2023/07/02
2023/06/26	Lorazepam	0.5 mg	Oral	Once daily at bedtime	2023/07/02
2023/06/30	Paliperidone ER	3 mg	Oral	Once daily	2023/07/02
2023/06/30	Mirtazapine	7.5 mg	Oral	Once daily at bedtime	2023/07/02

ER, extended-release; IV, intravenous; QHS, once daily at bedtime. The patient was hospitalized from June 6 to July 2, 2023. Clinical changes occurred after evening medications on June 21. Start/stop dates indicate the first and last day of each drug; frequency indicates daily doses. Only generic names are listed; brand names and indications are omitted.

## 3 Discussion

Delirium is an acute and typically fluctuating mental state characterized by reduced attention and additional cognitive deficits or altered levels of awareness (24). It can arise from various causes, including medication use (26), mood disorders with psychotic features (27), neurological conditions (28), and alcohol withdrawal (29).

After confirming the diagnosis, we implemented a combination therapy regimen, including antidepressants, benzodiazepines, and second-generation antipsychotics (30–33). We ruled out the possibility of other medications causing delirium for several reasons. First, the onset and resolution of the patient’s delirium symptoms were closely correlated with the administration and discontinuation of bupropion, suggesting that the delirium was likely directly associated with bupropion use. Second, reports of paliperidone and olanzapine inducing delirium or hallucinations are limited (34), bupropion is more commonly associated with delirium. Lastly, from a pharmacological perspective of these medications, buspirone, paliperidone, and olanzapine exert antipsychotic effects primarily by modulating dopamine D2 receptors and also influence 5-HT receptors, which helps improve mood and cognitive function (35–38). The use of these medications tends to stabilize the patient’s mental state rather than act as a precipitating factor for delirium. We therefore consider the patient’s visual hallucinations to be strongly associated with bupropion use. The unique clinical presentation of this patient following bupropion administration highlights a possible link between the medication and the observed psychiatric symptoms. The patient had no previous history of bupropion use or delirium. A score of 5 on the Chinese version of the Naranjo Adverse Drug Reaction Probability Scale suggests a probable adverse reaction to bupropion (25).

Several factors support the hypothesis that bupropion triggered the delirium. First, bupropion’s pharmacological profile, as it inhibits dopamine and norepinephrine reuptake, increases the concentration of these neurotransmitters in the brain (39). While this typically helps alleviate depressive symptoms, in some individuals, this significant shift may precipitate a state of central nervous system hyperexcitability, which can manifest as visual hallucinations. Secondly, due to the liver’s central role in drug metabolism and the patient’s history of chronic alcohol use, potential liver impairment cannot be ruled out potential liver damage (40). The impaired liver function could reduce drug clearance, extending the half-life of bupropion (41) and increasing dopamine accumulation risk. With bupropion and its active metabolite bupropion having half-lives of approximately 21 and 20 hours, respectively (42), the timing of hallucination onset and resolution aligned with bupropion use, further reinforcing the connection.

Activation of reward pathways suppresses the anti-reward system, and vice versa (43). The amygdala, particularly the basolateral amygdala, is a key node of the anti-reward system and regulates adaptive behavior through distributed neural networks. Animal and human studies indicate that bilateral amygdala lesions impair reward processing while preserving familiarity-based recognition (44, 45). Repeated exposure to psychoactive substances may weaken reward mechanisms and enhance anti-reward processes (46). However, the core features in this case—fluctuating attention and altered consciousness consistent with delirium—cannot be explained by reward–anti-reward imbalance alone. These symptoms likely reflect broader dysfunction of arousal and cognitive networks involving neurotransmitter dysregulation, neuroendocrine stress, and inflammation (47, 48). The rapid onset and resolution after bupropion suggest a functional mechanism, with prior amygdala damage acting as a vulnerability factor rather than the sole cause.

Additional factors may have contributed to the patient’s presentation. Although blood ethanol levels were negative upon admission and during the hallucination episodes, long-term alcohol use is known to induce neuroadaptation (49), which can involve elevated oxidative stress and reactive oxygen species (ROS) production during early withdrawal. This may induce excitotoxicity via increased excitatory neurotransmission and intracellular calcium levels (50), ultimately resulting in oxidative metabolic and neurotransmission disruption related to alcohol withdrawal, potentially contributing to delirium (29, 51).

Furthermore, the patient had undergone Bilateral Stereotactic Deep Brain Nucleus Lesioning Surgery, a neurosurgical intervention used for emotional regulation (52). Emerging evidence suggests that impaired amygdala function may be associated with psychotic features (53). The potential impacts of surgical intervention on the nervous system, metabolism, and blood-brain barrier integrity could heighten sensitivity to medications and their side effects in this patient (51), potentially intensifying bupropion’s effects on dopamine and norepinephrine, raising neurotransmitter levels, and precipitating adverse neuropsychiatric reactions (54).

Nevertheless, limitations remain in this case report. The severity of long-term alcohol use was assessed using the Michigan Alcohol Screening Test (MAST, score = 15), which evaluates chronic alcohol-related problems rather than acute withdrawal. Clinical observations during hospitalization and at delirium onset revealed no typical withdrawal features, such as tremor, diaphoresis, autonomic instability, agitation, or seizures. Based on this assessment, the patient met ICD-11 criteria for harmful alcohol use, rather than alcohol dependence or acute withdrawal syndrome. Given the use of multiple medications in situations where single antidepressant efficacy is limited, complex drug interactions may arise (55). Further studies are warranted to elucidate bupropion’s mechanisms and its potential interaction with other medications in exacerbating such risks. In addition, formal bedside delirium screening instruments, such as the Confusion Assessment Method (CAM), were not routinely administered at the time of symptom onset. Therefore, the diagnosis of delirium was established retrospectively based on detailed clinical observation and comprehensive chart review, which may limit diagnostic precision.

Personalized treatment planning is essential, as patients exhibit individual differences regarding disease manifestation, drug metabolism, and comorbidities. Standardized treatment approaches may not adequately address these individual needs. Through tailored therapeutic strategies, it is possible to select appropriate drugs, adjust doses, and avoid potential adverse effects, thereby enhancing therapeutic efficacy and patient adherence.

## 4 Conclusion

This report underscores the importance of individualized pharmacological treatment and patient management in individuals with a history of chronic alcohol use, potential liver impairment, and prior brain surgery. Specifically, for such patients, when treating depressive episodes in bipolar disorder, bupropion may be associated with an increased risk of hallucinations. Consequently, if hallucinations occur, clinicians should promptly assess the likelihood of adverse drug reactions and consider discontinuing bupropion to ensure patient safety and optimize treatment outcomes of the treatment plan.

## Data Availability

The original contributions presented in the study are included in the article/supplementary material. Further inquiries can be directed to the corresponding author.
